# Supplementary material for: Dishonesty as a signal of trustworthiness: Honesty-Humility and trustworthy dishonesty
Source: R Soc Open Sci. 2020 Oct 28;7(10):200685. doi: 10.1098/rsos.200685 (PMC7657889; doi:10.1098/rsos.200685)
Supplement: Supplemental Material [file rsos200685supp1.docx]

**Supplemental Material**

**Deviations from the preregistrations**

#### Study 1: the initial aims

The initial aim of Study 1 was to test how Honesty-Humility (Ashton & Lee, 2008) and Guilt Proneness (Tangney & Dearing, 2004) differ with regard to cheating for the sake of the trustor when offered full trust (preregistered as “High Vulnerability”) as compared to no trust (preregistered as “Low Vulnerability”; see, <https://osf.io/s2k59/?view_only=7ba46a5f2bdf45a7bc763011c1c346f6>; masked for review). The idea to explore the phenomena of trustworthy dishonesty arose after data collection when we observed that some participants engaged in trustworthy dishonesty as a response to being offered full trust by their trustor (see, Tables 1a-2b).

#### Studies 1-3: the reasoning behind the 10% threshold

In the preregistrations for both Studies 2 and 3 (see <https://osf.io/dtk93/?view_only=907fccbcc7b04bf694caa061ba83199e> and <https://osf.io/x8mz3/?view_only=7dc346f22ac4451892deec17035a2fcb>, respectively; masked for review), we planned to compute the proportion of individuals dishoneslty underreporting the income-maximizing outcome for the entire sample (Hypotheses 1 in both studies) and for the participants above and below the median of Honesty-Humility (Hypotheses 2 in both studies). These hypotheses were based on Study 1 where we observerd a significant proportion of such individuals in the Full Trust condition (*P* = 0.21; 95% CI [0.07; 0.35]). In addition, we observed that in the Full Trust condition, participants high in Honesty-Humility (above the median) were descriptively more likely to underreport the income-maximizing outcome (*P* = 0.33 [0.14; 0.51]) as compared to participants low in Honesty-Humility (below the median; *P* = 0.11 [0; 0.31]; *ΔG^2^* (1) = 2.73, *p* = .10).

However, in Studies 2 and 3, we did not manage to obtain similar results (see the “Confirmatory analyses” sections of Studies 2 and 3). After visually examining the data (see Figures 2 and 3), we concluded that although in Study 2 there was no underreporting of the income-maximizing outcome even among participants with the highest scores in Honesty-Humility, there was a small proportion of individuals who may have underreported this outcome in Study 3. The former is indicated by the 95% CI of the probability slope not falling below the expected probability of reporting the income maximizing outcome assuming full honesty (see Figure 2), while the latter is indicated by the entire 95% confidence interval of the probability slope falling entirely below the expected probability of reporting the income maximizing outcome assuming full honesty for the highest scores in Honesty-Humility (see Figure 3). Following this observation, we estimated that the lowest value of Honesty-Humility above which the 95% confidence interval of the probability in question falls below the expected probability (assuming full honesty), equals approximately 4.41 (*P* = 0.15; 95% CI [0.12; 0.16]). This indicates that for the participants with Honesty-Humility scores above or equal to 4.41 (which corresponds to approximately the top 10%)^[[1]](#footnote-1)^, the probability of reporting the income-maximizing outcome was lower than expected assuming full honesty. Because this proportion was much smaller than what was observed in Study 1 (where the probability in question was lower than expected for the top 67.37%; see Figure 3), we decided to apply the 10% threshold across all three studies. This allows us to make rather conservative conclusions about trustworthy dishonesty only with regard to the participants with the very highest scores in Honesty-Humility (rather than to the majority of participants, as it would be indicated in Study 1).


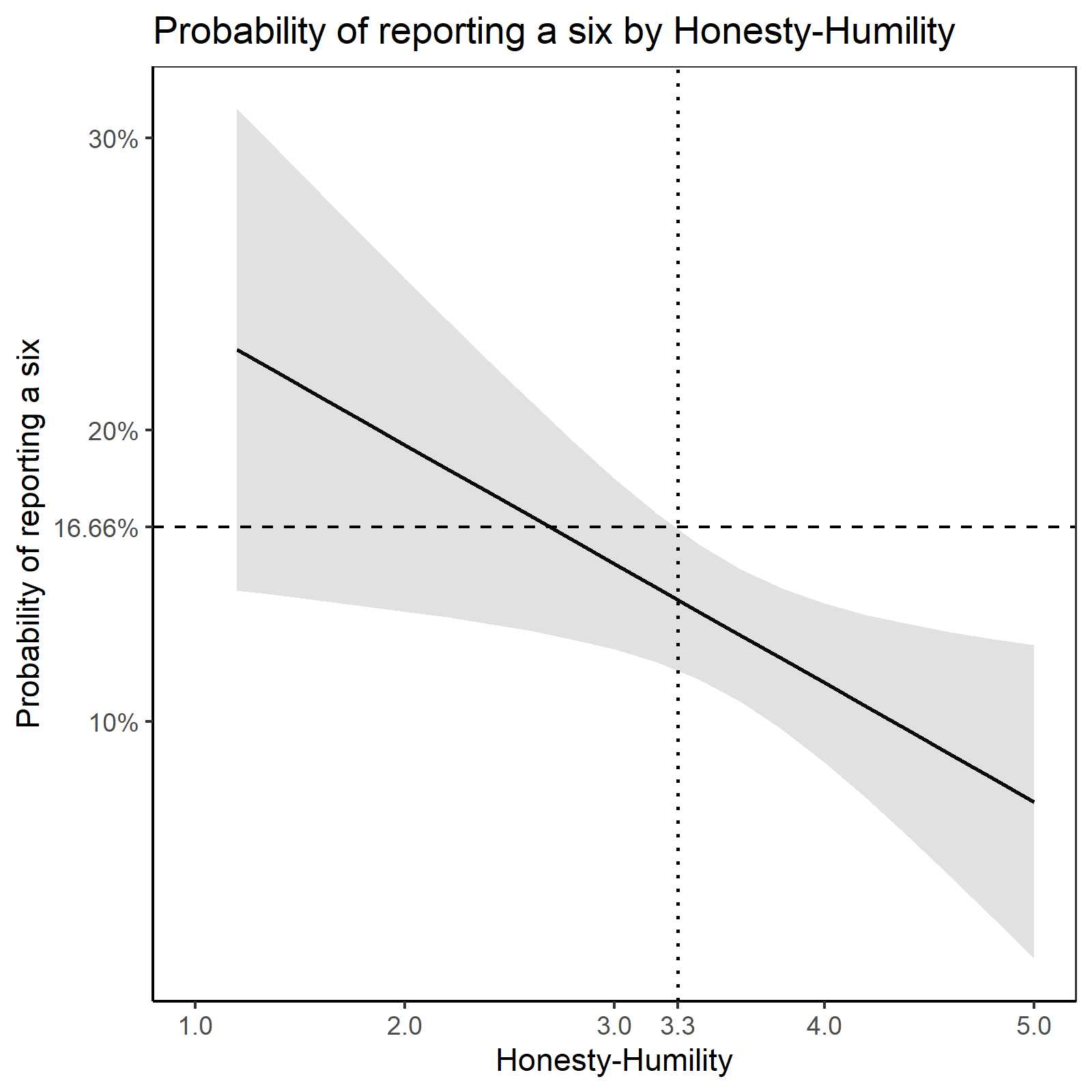


*Figure 1.* Probability of reporting the income maximizing outcome (six) by Honesty-Humility in Study 1. The dashed horizontal line indicates the expected probability of reporting the income maximizing outcome assuming full honesty. The gray ribbon illustrates the 95% confidence interval. The dotted vertical line indicates the lowest value of Honesty-Humility above which the 95% CI of the probability slope falls below the expected probability of reporting the income maximizing outcome assuming full honesty (3.3; *P* = 0.14; 95% CI [0.12; 0.165]). The value of Honesty-Humility is larger or equal to 3.3 for 67.37% of the participants. *N* = 1,713.


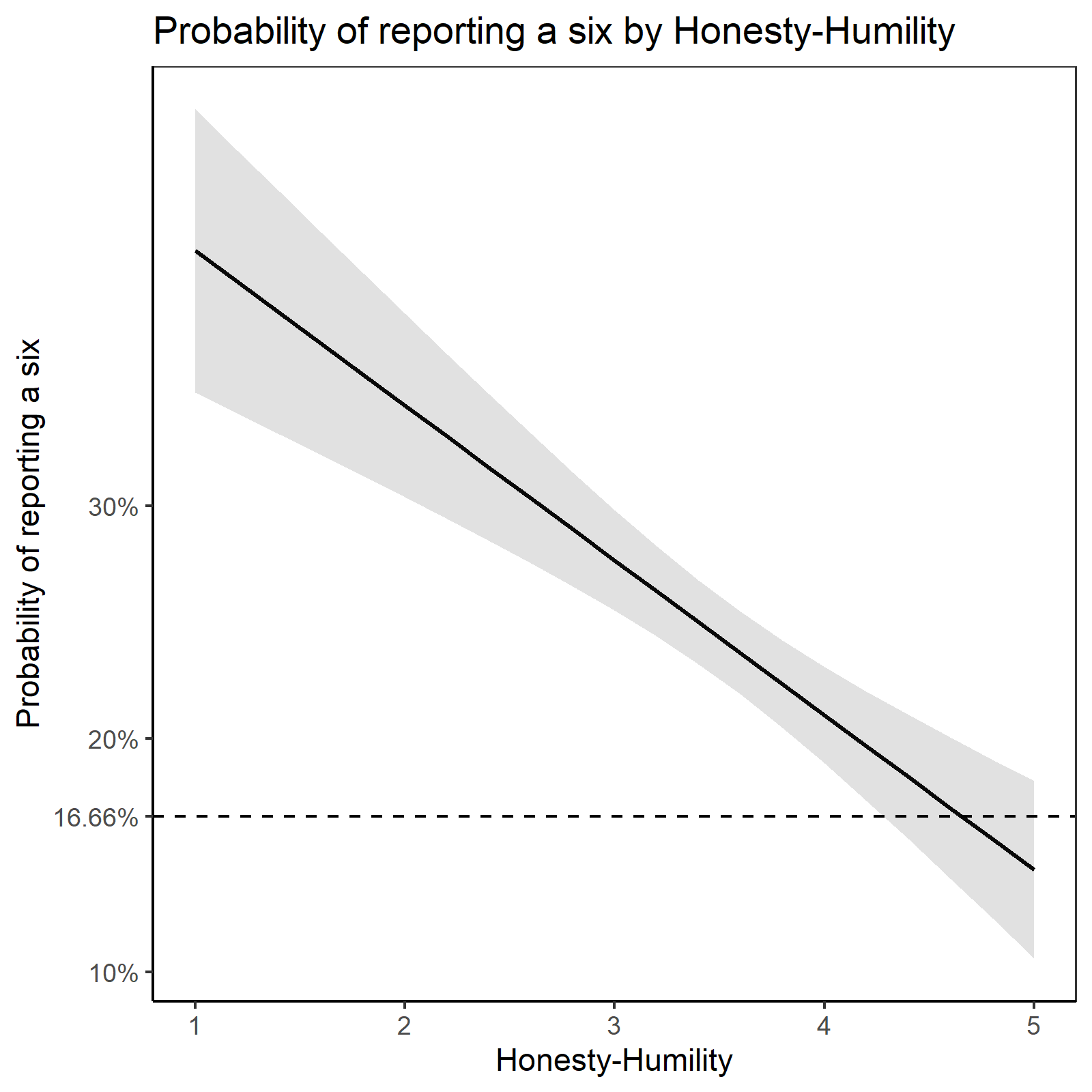


*Figure 2.* Probability of reporting the income maximizing outcome (six) by Honesty-Humility in Study 2. The dashed horizontal line indicates the expected probability of reporting the income maximizing outcome assuming full honesty. The gray ribbon illustrates the 95% confidence interval. The 95% CI does not fall below the expected probability of reporting the income maximizing outcome assuming full honesty. *N* = 2,230


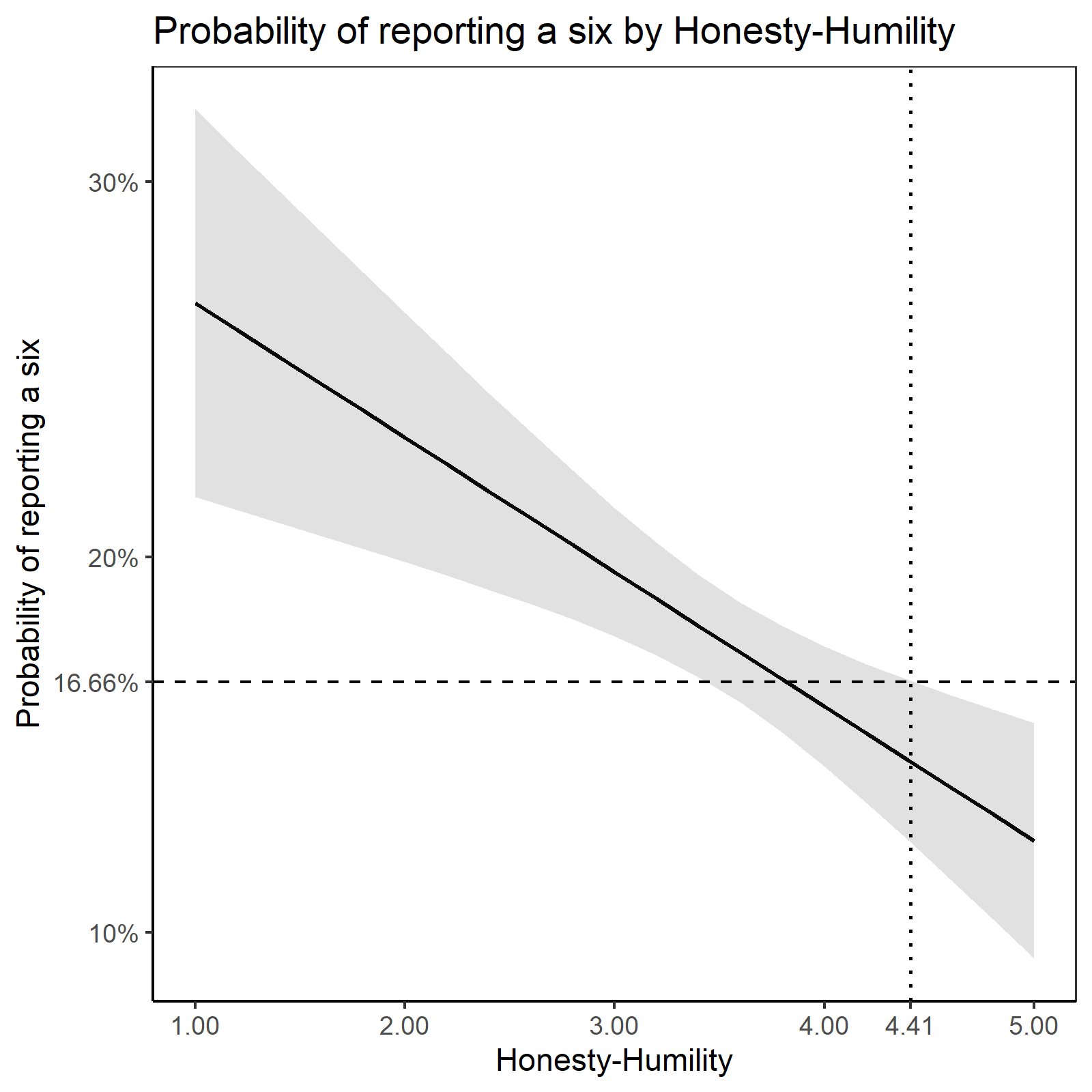


*Figure 3.* Probability of reporting the income maximizing outcome (six) by Honesty-Humility in Study 3. The dashed horizontal line indicates the expected probability of reporting the income maximizing outcome assuming full honesty. The gray ribbon illustrates the 95% confidence interval. The dotted vertical line indicates the lowest value of Honesty-Humility above which the 95% CI of the probability slope falls below the expected probability of reporting the income maximizing outcome assuming full honesty (4.41; *P* = 0.15, 95% CI [0.12, 0.16]). The value of Honesty-Humility is larger or equal to 4.41 for 9.40% of the participants. *N* = 3,137.

## Study 1

### Confirmatory analyses

The results of the pre-registered analyses are presented below.

- Hypothesis 1: One tailed one sample t-test indicates that the mean of reported numbers (*M* = 3.73; *SD* = 1.67) was higher than what was expected assuming full honesty (*M*  = 3.5; *t*(1,712) = 5.72; *p* < .001; one-tailed). The hypothesis was thus supported.
- Hypothesis 2: One tailed independent t-test indicates that the mean of reported numbers in the Full Trust condition (*M* = 3.51; *SD* = 1.61) was lower than the mean of reported numbers in the No Trust condition (*M* = 3.95; *SD* = 1.71; *t*(1,706.8) = -5.45; *p* < .001; one-tailed). The hypothesis was thus supported.
- Hypothesis 3: Multiple linear regression indicates that there was no significant interaction between Guilt Proneness and condition (*B* = .05; 95% CI [-0.05; 0.14]; *p*  = .170; one-tailed). The hypothesis was thus not supported.
- Hypothesis 4: Multiple linear regression indicates that there was no significant interaction between Honesty-Humility and condition (*B* = -.02; 95% CI [-0.11; 0.07]; *p*  = .339; one-tailed). In addition, equivalence testing indicates that the 95% confidence interval of the obtained effect size (*f^2^* = 0; 95% CI [-0; 0]) falls entirely within the assumed equivalence bound of *f^2^* = |0.01|. The hypothesis was thus supported.
- Hypothesis 5: Multiple linear regression indicates that both Honesty-Humility and Guilt Proneness significantly predicted dishonesty in the Full Trust condition (*B* = -.14; 95% CI [-0.24; -0.03]; *p*  = .007; one tailed, and *B* = -.11; 95% CI [-0.24; -0.003]; *p*  = .021; one-tailed, respectively). The hypothesis was thus supported.

### Over/underreporting and Honesty-Humility (median splits)

Table 1a

*Study 1 – Full trust condition*

| Die roll | Proportion of overreporting dishonest individuals | | Proportion of underreporting dishonest individuals | |
| --- | --- | --- | --- | --- |
|  | high HH | low HH | high HH | low HH |
| 1 | 0.01 [0; 0.05] | 0.01 [0; 0.05] | 0.04 [0; 0.19] | 0.16 [0; 0.36] |
| 2 | 0.01 [0; 0.05] | 0.01 [0; 0.05] | 0.16 [0; 0.35] | **0.30 [0.10; 0.48]** |
| 3 | 0.02 [0; 0.06] | 0.03 [0; 0.08] | 0.04 [0; 0.22] | 0.05 [0; 0.22] |
| 4 | **0.07 [0.03; 0.12]** | **0.07 [0.02; 0.12]** | 0.04 [0; 0.22] | 0.05 [0; 0.21] |
| 5 | 0.01 [0; 0.05] | 0.01 [0; 0.06] | 0.05 [0; 0.20] | 0.05 [0; 0.22] |
| 6 | 0.01 [0; 0.04] | 0.01 [0; 0.04] | **0.33 [0.14; 0.51]** | 0.11 [0; 0.31] |

*Note.* 95% confidence intervals of the values in bold do not include zero. HH = Honesty-Humility, high HH = values above the median, low HH = values below the median. *N* = 808.

Table 1b

*Study 1 – No trust condition*

| Die roll | Proportion of overreporting  dishonest individuals | | Proportion of underreporting  dishonest individuals | |
| --- | --- | --- | --- | --- |
|  | high HH | low HH | high HH | low HH |
| 1 | 0.01 [0; 0.04] | 0.01 [0; 0.05] | 0.09 [0; 0.29] | **0.35 [0.16; 0.54]** |
| 2 | 0.01 [0; 0.05] | 0.01 [0; 0.04] | **0.30 [0.11, 0.48]** | **0.41 [0.21; 0.58]** |
| 3 | 0.01 [0; 0.04] | 0.01 [0; 0.03] | 0.09 [0; 0.28] | **0.27 [0.07; 0.46]** |
| 4 | 0.01 [0; 0.05] | 0.01 [0; 0.03] | 0.04 [0; 0.21] | 0.11 [0; 0.30] |
| 5 | **0.06 [0.01; 0.11]** | **0.10 [0.05; 0.14]** | 0.04 [0; 0.21] | 0.04 [0; 0.23] |
| 6 | 0.03 [0; 0.07] | **0.13 [0.08; 0.16]** | 0.04 [0; 0.21] | 0.04 [0; 0.21] |

*Note.* 95% confidence intervals of the values in bold do not include zero. HH = Honesty-Humility, high HH = values above the median, low HH = values below the median. *N* = 804.

**Over/underreporting and Honesty-Humility (top and bottom 25%)**

Table 2a

*Study 1 – Full trust condition,*

| Die roll | Proportion of overreporting dishonest individuals | | Proportion of underreporting dishonest individuals | |
| --- | --- | --- | --- | --- |
|  | high HH | low HH | high HH | low HH |
| 1 | 0.03 [0; 0.08] | 0.01 [0; 0.06] | 0.06 [0; 0.30] | **0.38 [0.13; 0.63]** |
| 2 | 0.01 [0; 0.05] | 0.01 [0; 0.06] | 0.18 [0; 0.49] | 0.15 [0; 0.41] |
| 3 | 0.04 [0; 0.10] | 0.01 [0; 0.06] | 0.06 [0; 0.27] | 0.06 [0; 0.30] |
| 4 | **0.09 [0.03; 0.18]** | **0.08 [0.01; 0.15]** | 0.06 [0; 0.27] | 0.07 [0; 0.30] |
| 5 | 0.01 [0; 0.05] | 0.02 [0; 0.08] | 0.12 [0; 0.35] | 0.07 [0; 0.30] |
| 6 | 0.01 [0; 0.07] | 0.01 [0; 0.06] | **0.53 [0.28; 0.75]** | 0.07 [0; 0.30] |

*Note.* 95% confidence intervals of the values in bold do not include zero. HH = Honesty-Humility, high HH = values within the top 25%, low HH = values within the bottom 25%. *N* = 426.

Table 2b

*Study 1 – No trust condition*

| Die roll | Proportion of overreporting  dishonest individuals | | Proportion of underreporting  dishonest individuals | |
| --- | --- | --- | --- | --- |
|  | high HH | low HH | high HH | low HH |
| 1 | 0.02 [0; 0.07] | 0.01 [0; 0.06] | 0.06 [0; 0.27] | **0.42 [0.16; 0.67]** |
| 2 | 0.01 [0; 0.06] | 0.01 [0; 0.06] | **0.41 [0.16; 0.64]** | **0.47 [0.22; 0.67]** |
| 3 | 0.01 [0; 0.06] | 0.01 [0; 0.06] | 0.17 [0; 0.41] | 0.15 [0; 0.41] |
| 4 | 0.01 [0; 0.06] | 0.01 [0; 0.06] | 0.09 [0; 0.36] | 0.33 [0.08; 0.58] |
| 5 | **0.07 [0.01; 0.14]** | 0.07 [0.01; 0.15] | 0.07 [0; 0.27] | 0.06 [0; 0.30] |
| 6 | 0.05 [0; 0.11] | **0.20 [0.12; 0.27]** | 0.06 [0; 0.3] | 0.06 [0; 0.30] |

*Note.* 95% confidence intervals of the values in bold do not include zero. HH = Honesty-Humility, high HH = values within the top 25%, low HH = values within the bottom 25%. *N* = 428.

## Study 2

**Confirmatory analyses**

In Study 2 we have preregistered two Hypotheses. First of all, please note that we have not achieved the sample size we planned (*N* = 3,020) due to not enough participants completing the second wave of the study. Therefore these results should be interpreted with caution. The pre-registered analyses are presented below:

- Hypothesis 1: We planned to test for the occurrence of underreporting of the income-maximizing outcome (six) in the entire sample. We found that the proportion of individuals dishonestly underreporting this outcome was insignificant *P* = 0.02 [0; 0.09]. The hypothesis was thus not supported.
- Hypothesis 2: We planned to test for the occurrence of underreporting of the income maximizing outcome (six) in people high and low in Honesty-Humility. In this realm we preregistered two analyses:
  - Analysis 1: Logistic regression predicting if participants reported the income-maximizing outcome with Honesty-Humility. We planned calculate the percentage of participants for whom the 95% confidence interval of the probability of reporting the income-maximizing outcome falls below the theoretical probability of reporting the income-maximizing outcome assuming full honesty. As can be seen in Figure 2, the entire 95% CI of the probability of reporting the income-maximizing outcome does not fall below the theoretical threshold. The hypothesis was thus not supported.
  - Analysis 2: We planned to test for the occurrence of underreporting of the income-maximizing outcome (six) in participants high (above the median) and low (below the median) in Honesty-Humility, which also turned out to be insignificant (*P* = 0.03 [0; 0.14]; *P* = 0.03 [0; 0.13], respectively). The hypothesis was thus not supported. Please note that we could not include Honesty-Humility as a continuous variable, because currently multiTree does not support continuous variables (Moshagen, 2010).
- .

**Over/underreporting and Honesty-Humility (median splits)**

Table 3

*Study 2*

| Die roll | Proportion of overreporting dishonest individuals | | Proportion of underreporting dishonest individuals | |
| --- | --- | --- | --- | --- |
|  | high HH | low HH | high HH | low HH |
| 1 | 0.01 [0; 0.03] | 0.01 [0; 0.03] | **0.49 [0.39; 0.60]** | **0.52 [0.43; 0.62]** |
| 2 | 0.01 [0; 0.03] | 0.01 [0; 0.03] | **0.46 [0.35; 0.57]** | **0.53 [0.44; 0.63]** |
| 3 | **0.03 [0.003; 0.06]** | 0.01 [0; 0.03] | 0.03 [0; 0.13] | **0.21 [0.09; 0.33]** |
| 4 | **0.08 [0.05; 0.11]** | **0.04 [0.01; 0.07]** | 0.03 [0; 0.13] | 0.03 [0; 0.13] |
| 5 | **0.05 [0.02; 0.08]** | **0.06 [0.03; 0.09]** | 0.03 [0; 0.14] | 0.03 [0; 0.13] |
| 6 | **0.04 [0.01; 0.07]** | **0.15 [0.12; 0.18]** | 0.03 [0; 0.14] | 0.03 [0; 0.13] |

*Note.* 95% confidence intervals of the values in bold do not include zero. HH = Honesty-Humility, high HH = values above the median, low HH = values below the median. *N* = 2,118

.

**Over/underreporting and Honesty-Humility (top and bottom 25%)**

Table 4

*Study 2*

| Die roll | Proportion of overreporting dishonest individuals | | Proportion of underreporting dishonest individuals | |
| --- | --- | --- | --- | --- |
|  | high HH | low HH | high HH | low HH |
| 1 | 0.01 [0; 0.04] | 0.01 [0; 0.04] | **0.53 [0.39; 0.66]** | **0.55 [0.40; 0.69]** |
| 2 | 0.01 [0; 0.04] | 0.01 [0; 0.04] | **0.43 [0.27; 0.57]** | **0.57 [0.44; 0.70]** |
| 3 | 0.01 [0; 0.05] | 0.01 [0; 0.04] | 0.04 [0; 0.18] | **0.30 [0.13; 0.44]** |
| 4 | **0.08 [0.04; 0.12]** | 0.02 [0; 0.06] | 0.04 [0; 0.17] | 0.04 [0; 0.18] |
| 5 | **0.07 [0.03; 0.11]** | **0.07 [0.04; 0.12]** | 0.04 [0; 0.18] | 0.04 [0; 0.19] |
| 6 | 0.03 [0; 0.07] | **0.19 [0.14; 0.24]** | 0.04 [0; 0.18] | 0.04 [0; 0.18] |

*Note.* 95% confidence intervals of the values in bold do not include zero. HH = Honesty-Humility, high HH = values within the top 25%, low HH = values within the bottom 25%. *N* = 1,116.

## Study 3

**Confirmatory analyses**

In Study 3, we preregistered identical Hypotheses as in Study 2. The pre-registered analyses are presented below:

- Hypothesis 1: The proportion of participants who dishonestly underreported the income-maximizing outcome (six) in the entire sample was insignificant (*P* = 0.02 [0; 0.08]). The hypothesis was thus not supported.
- Hypothesis 2:
  - Analysis 1: As can be seen in Figure 3, the entire 95% CI of the probability of reporting the income-maximizing outcome falls below the theoretical threshold for participants who scored 4.41 or higher in Honesty-Humility (which covers participants in the approximately top 10% scores in Honesty-Humility). The hypothesis was thus supported using this analysis.
  - Analysis 2: The proportion of participants who dishonestly underreported the income-maximizing outcome (six) was insignificant both among participants with Honesty-Humility scores above (*P* = 0.07 [0; 0.17]) and below (0.02 [0; 0.12]) the median Honesty-Humility. The hypothesis was thus not supported using this analysis.

**Over/underreporting and Honesty-Humility (median splits)**

Table 5

*Study 3 – Full trust condition, median split*

| Die roll | Proportion of overreporting dishonest individuals | | Proportion of underreporting dishonest individuals | |
| --- | --- | --- | --- | --- |
|  | high HH | low HH | high HH | low HH |
| 1 | 0.005 [0; 0.02] | 0.005 [0; 0.02] | **0.21 [0.10; 0.32]** | **0.44 [0.35; 0.52]** |
| 2 | 0.01 [0; 0.02] | 0.005 [0; 0.02] | **0.27 [0.16; 0.37]** | **0.30 [0.20; 0.30]** |
| 3 | **0.03 [0.01; 0.06]** | 0.02 [0; 0.05] | 0.02 [0; 0.12] | 0.02 [0; 0.11] |
| 4 | **0.07 [0.05; 0.10]** | **0.09 [0.07; 0.12]** | 0.02 [0; 0.12] | 0.02 [0; 0.12] |
| 5 | 0.01 [0; 0.03] | 0.005 [0; 0.02] | 0.02 [0; 0.11] | 0.02 [0; 0.11] |
| 6 | 0.005 [0; 0.02] | **0.03 [0.01; 0.06]** | 0.07 [0; 0.17] | 0.02 [0; 0.12] |

*Note.* 95% confidence intervals of the values in bold do not include zero. HH = Honesty-Humility, high HH = values above the median, low HH = values below the median. *N* = 2,967.

**Over/underreporting and Honesty-Humility (top and bottom 25%)**

Table 4

*Study 3 – Full trust condition, 25% split*

| Die roll | Proportion of overreporting dishonest individuals | | Proportion of underreporting dishonest individuals | |
| --- | --- | --- | --- | --- |
|  | high HH | low HH | high HH | low HH |
| 1 | 0.01 [0; 0.03] | 0.01 [0; 0.03] | **0.15 [0.01; 0.30]** | **0.47 [0.35; 0.59]** |
| 2 | 0.01 [0; 0.03] | 0.01 [0; 0.03] | **0.25 [0.11; 0.40]** | **0.31 [0.17; 0.44]** |
| 3 | **0.04 [0.01; 0.08]** | **0.03 [0.001; 0.07]** | 0.03 [0; 0.17] | 0.03 [0; 0.16] |
| 4 | **0.06 [0.03; 0.10]** | **0.09 [0.05; 0.12]** | 0.03 [0; 0.16] | 0.03 [0; 0.14] |
| 5 | 0.01 [0; 0.04] | 0.01 [0; 0.03] | 0.03 [0; 0.17] | 0.04 [0; 0.17] |
| 6 | 0.01 [0; 0.03] | **0.04 [0.01; 0.08]** | 0.14 [0; 0.29] | 0.03 [0; 0.15] |

*Note.* 95% confidence intervals of the values in bold do not include zero. HH = Honesty-Humility, high HH = values within the top 25%, low HH = values within the bottom 25%. *N* = 1,568.

## Age and gender differences between people in the top 10% in HH and the remaining 90%

- Study 1
  - Age: top 10%: *M_age_* = 42.22; remaining 90%: *M_age_* = 35.70; *t*(209.43) = 6.51; *p* < .001
  - Gender: top 10%: 83.04% female; remaining 90%: 67.77% female
- Study 2
  - Age: top 10%: *M_age_* = 44.62; remaining 90%: *M_age_* = 37.58; *t*(265.74) = 7.82; *p* < .001
  - Gender: top 10%: 62.16% female; remaining 90%: 50.80% female
- Study 3
  - Age: top 10%: *M_age_* = 42.30; remaining 90%: *M_age_* = 35.55; *t*(370.65) = 8.66; *p* < .001
  - Gender: top 10%: 79.30% female; remaining 90%: 66.91% female

1. More specifically, it corresponds to 9.40%. However, we assume the value of 10% for simplicity. [↑](#footnote-ref-1)
